# Flexible BaTiO_3_-PDMS Capacitive Pressure Sensor of High Sensitivity with Gradient Micro-Structure by Laser Engraving and Molding

**DOI:** 10.3390/polym15153292

**Published:** 2023-08-03

**Authors:** Jiayi Li, Shangbi Chen, Jingyu Zhou, Lei Tang, Chenkai Jiang, Dawei Zhang, Bin Sheng

**Affiliations:** 1 School of Optical Electrical and Computer Engineering, University of Shanghai for Science and Technology, Shanghai 200093, China; 2035051414@st.usst.edu.cn (J.L.); 2133305789@st.usst.edu.cn (J.Z.); leitangstark@foxmail.com (L.T.); 212180323@st.usst.edu.cn (C.J.); dwzhang@usst.edu.cn (D.Z.); 2 Shanghai Key Laboratory of Modern Optical Systems, Engineering Research Center of Optical Instruments and Systems, Shanghai 200093, China; 3 Inertial Technology Division, Shanghai Aerospace Control Technology Institute, Shanghai 201109, China; chensb@mail.ustc.edu.cn

**Keywords:** flexible capacitance pressure sensor, gradient micro-structure, polymer, barium titanate, laser engraving

## Abstract

The significant potential of flexible sensors in various fields such as human health, soft robotics, human–machine interaction, and electronic skin has garnered considerable attention. Capacitive pressure sensor is popular given their mechanical flexibility, high sensitivity, and signal stability. Enhancing the performance of capacitive sensors can be achieved through the utilization of gradient structures and high dielectric constant media. This study introduced a novel dielectric layer, employing the BaTiO_3_-PDMS material with a gradient micro-cones architecture (GMCA). The capacitive sensor was constructed by incorporating a dielectric layer GMCA, which was fabricated using laser engraved acrylic (PMMA) molds and flexible copper-foil/polyimide-tape electrodes. To examine its functionality, the prepared sensor was subjected to a pressure range of 0–50 KPa. Consequently, this sensor exhibited a remarkable sensitivity of up to 1.69 KPa^−1^ within the pressure range of 0–50 KPa, while maintaining high pressure-resolution across the entire pressure spectrum. Additionally, the pressure sensor demonstrated a rapid response time of 50 ms, low hysteresis of 0.81%, recovery time of 160 ms, and excellent cycling stability over 1000 cycles. The findings indicated that the GMCA pressure sensor, which utilized a gradient structure and BaTiO_3_-PDMS material, exhibited notable sensitivity and a broad linear pressure range. These results underscore the adaptability and viability of this technology, thereby facilitating enhanced flexibility in pressure sensors and fostering advancements in laser manufacturing and flexible devices for a wider array of potential applications.

## 1. Introduction

In recent times, the pressure sensor garnered significant attention due to its wide-ranging applications in diverse fields such as soft robotics [1,2], human–machine interaction [3], electronic skin [4,5,6], tactile and touch sensing applications [7,8], contactless sensing [9], and information communication [10]. Presently, pressure sensors can be primarily categorized into resistance [11,12,13], capacitance [11,14,15], piezoelectric [11,16], and triboelectric types [11,17] based on distinct transduction mechanisms. Capacitive sensors are favored among these types due to their simple structure, ease of fabrication, low-energy consumption, and ability to precisely modify device design through analysis of the governing equation. In terms of performance, they demonstrate notable attributes such as high sensitivity and rapid response times. Additionally, these sensors have been proven to replicate the sensing behavior of human skin, encompassing strain sensitivity, pressure detection, and proximity sensing [18]. Furthermore, capacitive pressure sensors exhibit low power consumption and can be engineered to be unaffected by temperature variations [18,19,20,21,22]. Consequently, they are deemed attractive. To cater to various applications, capacitive sensors are required to exhibit both high sensitivity and a wide linear range. The sensitivity of traditional capacitive sensors utilizing solid dielectric layers is hindered by their restricted deformation capacity [23,24,25]. In order to enhance sensitivity, extensive research has been conducted on diverse micro-structures such as micro-spheres [26], micro-pillars [27], porous structures [28], micro-pyramids [29], nanoparticles [30,31,32], and micro-array structures [33,34]. However, it has been observed that these micro-structures primarily operate within the low-pressure range, thereby diminishing their sensitivity in the high-pressure range and consequently limiting the linear range [21,35,36,37,38,39]. Previous studies indicated that the utilization of gradient structures can effectively enhance linearity and substantially augment sensitivity [21]. It is important to acknowledge that the sensitivity of sensor components featuring microstructure effectively trades off against hysteresis. According to scholarly research, the implementation of a random distribution of pixels featuring gradient structures has been found to effectively diminish interface adhesion [40]. Consequently, this approach enables the sensor to sustain high sensitivity levels while minimizing hysteresis.

A flexible material possessing a high permittivity is crucial for the development of flexible capacitive sensors and charge storage devices. Currently, ferroelectric polymer materials, including poly-(vinylidene fluoride)(PVDF) and poly-(vinylidene fluoride- trifluoroethylene)[P(VDF-TrFE)], have been successfully employed to achieve high permittivity. Nevertheless, these polymers exhibit temperature instability and can lead to device corrosion due to the formation of hydrogen fluoride [41]. Several studies investigated the advancement of composite materials possessing superior dielectric properties in order to address the aforementioned constraints of organic polymers. The incorporation of ceramic fillers exhibiting high permittivity represents a prevalent approach for enhancing the dielectric permittivity of polymers [42,43]. For instance, flexible materials like polydimethylsiloxane (PDMS) were combined with Pb(Zr, Ti)O_3_ and MXene(Ti_3_C_2_T_x_) to fabricate dielectric layers [25,43]. The dielectric properties of these materials are influenced by the attributes of the constituents, the morphology and size of the additives, and the concentration of the additive [41]. BaTiO_3_ is a material possessing high permittivity and readily accessible [44]; thus, we opted for BaTiO_3_ powder blended with PDMS as the material for fabricating a flexible dielectric layer.

The dielectric layer of flexible pressure sensors is typically fabricated through the primary mold method, which traditionally involves lithography or the utilization of natural molds such as lotus leaves and petals [45,46,47,48,49,50]. However, the photo-lithography manufacturing process is intricate, expensive, and time-consuming, while the micro-structured surface created with natural molds is uneven and lacks controllable aspect ratios. In order to overcome these constraints, Valliammai Palaniappan et al. conducted a study to ascertain the viability of employing a laser-assisted engraving technique for the fabrication of PDMS dielectric layers. This method proved to be relatively uncomplicated, user-friendly, and time-efficient, requiring minimal preparation time [46,51,52,53]. Furthermore, during the laser-assisted production of the primary mold, the aspect ratio of the model can be readily manipulated by adjusting the power and scanning speed of the laser beam [46,54].

This study introduces a novel approach to capacitive pressure sensing by utilizing a BaTiO_3_-PDMS dielectric layer with a gradient micro-cones architecture (GMCA). The GMCA is achieved by laser carving a micro-cones hole array with varying heights on a black acrylic plate, which serves as a template for the deposition of the BaTiO_3_-PDMS dielectric layer. The pressure sensor is formed by sandwiching the dielectric layer between flexible electrodes composited with copper foil and polyimide tape. Consequently, the sensor exhibits a notable sensitivity of up to 1.69 KPa^−1^ within the pressure range of 0–50 KPa, ensuring the preservation of high pressure-resolution throughout the entire pressure spectrum. Additionally, the sensor demonstrates a rapid response time of 50 ms, minimal hysteresis of 0.81%, swift recovery time of 160 ms, and exceptional cycling stability over 1000 cycles. Consequently, this sensor possesses the capability to serve as a dependable motion recorder for comprehensive detection of physiological signals, including pulse, sound vibration, and joint flexion, among others.

## 2. Materials and Methods

### 2.1. Chemicals and Materials

A main mold was manufactured using a black poly (methyl methacrylate) (PMMA) with a thickness of approximately 5mm, sourced from Tian Gong company in Zhenjiang, Jiangsu, China. The barium titanate powder, with a particle size smaller than 3 μm, was obtained from Macklin company in Shanghai, China. The dielectric layers were manufactured using PDMS (SYLGARD 184 Silicone) from the DOW Chemical company in the Midland, MI, USA. The bonding process utilized ecoflex 00-30 from SMOOTH-ON Company in the Macungie, PA, USA, which included liquid A and liquid B. The flexible electrodes were manufactured using copper foil sourced from Zhengying Company in Anhui, China, and polyimide tape from Ubisoft Corporation, Hangzhou, Zhe Jiang, China.

### 2.2. Sensor Fabrication

**Figure 1:**
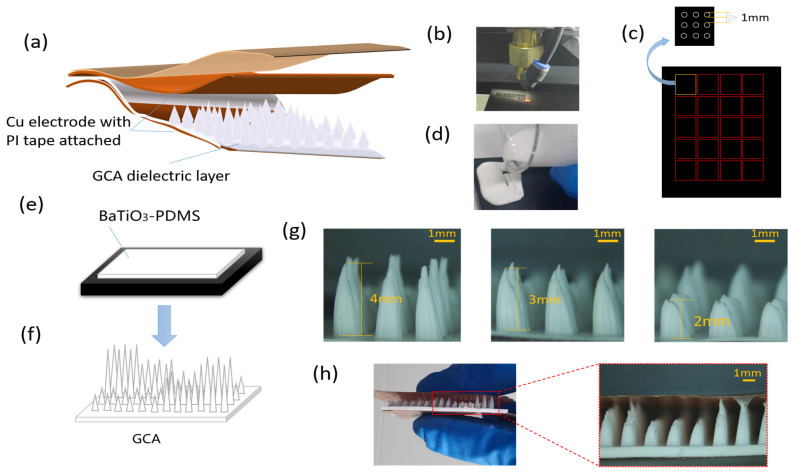
The manufacturing process of the pressure sensors. (**a**) Schematic diagram of the overall structure of the sensor (only some micro-structure pixels are shown in the figure). (**b**) Laser engraved hole array on the black acrylic board. (**c**) The main module. (**d**) The BaTiO_3_-PDMS was poured onto the acrylic main mold, and then it was vacuumed. (**e**) Heat at 60 °C for about one hour and wait for the dielectric layer to form. (**f**) The BaTiO_3_-PDMS dielectric layer GMCA was peeled-off from the main molds. (**g**) Digital microscope images and their heights of A, B, C three types of pixels. (**h**) The side view of the pressure sensor (PS4).

The sensor was comprised of two flexible electrode plates and a dielectric with a gradient micro-structure in the center (Figure 1a). The manufacturing process of a rectangular pressure sensor measuring 30 × 25 mm is depicted in Figure 1. Using AutoCAD™, a 3 × 3 array consisting of circles with a diameter of 1 mm was designed and then imported into the laser machine (K3020, Julong Laser Co., Ltd., Liaocheng, Shandong, China). The formation of the micro-cone exhibited a strong correlation with the laser power. The cross section intensity of the laser adhered to a Gaussian distribution, which played a significant role in the genesis of micro-cones [55,56]. As the laser ablation process advanced on the board, the gradual decoking resulted in a progressive decrease in the critical point’s radius, where the laser-induced ablation occurred, ultimately leading to the emergence of the micro-cone. Initially, a scanning speed of 100 mm/s was employed, along with a specific amount of light power, to carve a hole array consisting of 9 circular holes on the acrylic plate (Figure 1b). Next, the exit position of the laser beam and the light power should be adjusted to proceed with the creation of another hole array. This process should be repeated until a main mode consisting of 20 hole arrays, arranged in a 5 × 4 configuration, is achieved. The resulting main module, denoted as M, is depicted in Figure 1c. To prepare the PDMS, the elastomer base and hardener should be mixed in a ratio of 10:1, followed by thorough stirring with a glass rod for approximately 20 min. Subsequently, the obtained PDMS should be mixed with BaTiO_3_ powder in a mass ratio of 10:1 to obtain BaTiO_3_-PDMS, ensuring even distribution of the components. The mixed BaTiO_3_-PDMS composite was subjected to vacuum treatment using a vacuum machine (DZF-6053, Yong Guangming, Beijing, China) for approximately 30 min to eliminate any surface bubbles. Subsequently, the BaTiO_3_-PDMS composite was poured onto the acrylic main mold M (Figure 1d) and subjected to vacuum treatment for an additional 30 min. The vacuum machine was then heated to 60 °C for approximately one hour to facilitate curing (Figure 1e). Following the curing process, the BaTiO_3_-PDMS dielectric layer GMCA was carefully removed from the main molds M (Figure 1f). It is noteworthy that the utilization of lasers can be enhanced more effectively by employing black acrylic panels as opposed to conventional transparent acrylic panels, resulting in a higher aspect ratio of the tapered hole at equivalent optical power levels.

The electrode was composed of a copper foil that possessed adhesive on one side and was manufactured to have a thickness of 5 μm. Positioned above the dielectric layer, the lower surface of the micro-cone exhibited a seamless and uninterrupted plane, while the upper portion of the cone featured spaced-out spikes. The copper foil electrode can be affixed directly onto the smooth layer, while the spike tips can be gently positioned downwards onto the copper foil coated with a thin layer of ecoflex. This ecoflex substance was created by combining part A and part B in equal proportions of 1:1, followed by a vacuum process. Simultaneously, for the purpose of enhancing the electrode’s stability, it was possible to affix a layer of polyimide tape (thickness of 0.1 mm) onto the external surface of the electrode, thereby reducing the susceptibility of the copper foil to deformation. Following the curing of ecoflex, a capacitive sensor comprising two copper foil electrodes and a dielectric layer in between can be achieved.

To establish the gradient structure, the set layer power was modified, resulting in varying hole arrays corresponding to the layer power. In this experimental study, the nomenclature A, B, and C was assigned to the hole arrays based on their respective heights, with A representing the highest and C denoting the lowest. Considering the fabrication error of ~0.3 mm for laser engraving processing, the determination of the heights of three distinct micro cone structures (A, B, and C) was conducted to facilitate the establishment of gradient structures. Subsequently, the distance between the sensor electrode plates was ascertained. The composition of a GMCA, comprising multiple hole arrays, can be succinctly represented as AaBbCc, where the lowercase letters serve as subscripts indicating the number of hole arrays within the GMCA structure. Three arrays of holes with varying heights were randomly distributed on the main mode. The stochastic arrangement of pixels exhibiting varying heights facilitates the compatibility of the pressure sensor with diverse pixel configurations [41]. In order to demonstrate the significance of gradient structure and investigate the values of parameters a, b, and c, we also fabricated dielectric layers without gradient structure, which exhibited a highly uniform distribution of micro-cones architecture (MCA). The production method described above resulted in the creation of five dielectric layers, along with their respective parameters, as presented in Table 1. The images captured using a high-resolution digital microscope can be observed in Figure 1g, while the side view of the pressure sensor (PS4) that was fabricated is depicted in Figure 1h.

### 2.3. Experiment Setup

**Figure 2:**
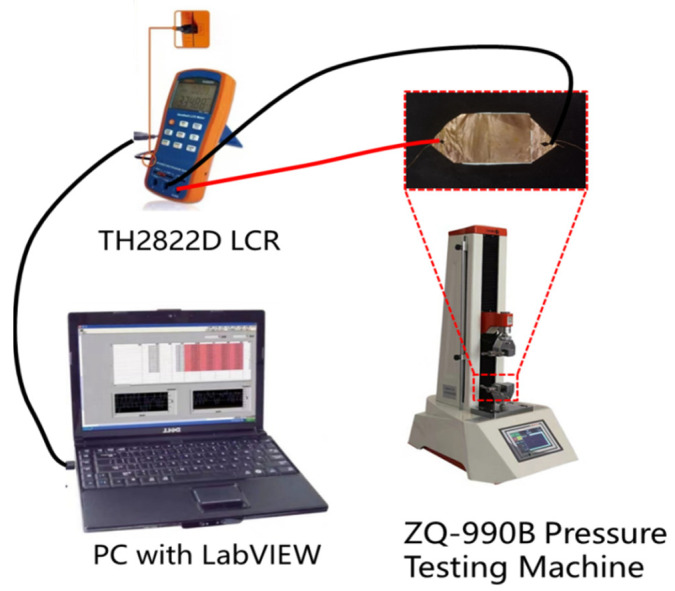
Experiment setup. The sensor was placed on the pressure testing platform and was connected to the LCR instrument, which was connected to the computer. When the pressure testing machine starts, LCR can measure the capacitance of the capacitor and upload it to the computer to record the data.

The experimental setup is depicted in Figure 2. The sensor was positioned on the pressure tester platform (Zhiqu company, Guangzhou, Guangdong, China) and subjected to a pressure range of 0–50 Kpa. The two electrodes of the capacitance sensor were connected to the digital bridge (LCR) instrument of Company Tonghui (Changzhou, Jiangsu, China) in order to measure the sensor’s capacitance response to varying pressure. A computer was linked to the LCR meter through a USB connection, facilitating post-processing and data analysis. All experiments were conducted under ambient room temperature conditions.

## 3. Results and Discussion

### 3.1. Capacitive Pressure Sensor Response

The working principle of capacitive pressure sensors can be explained by Equation (1) [21]:

(1) $$C=\frac{\varepsilon_{0}\varepsilon_{r}A}{d}$$

where *C*, *ε_r_*, *ε*_0_, *A*, and *d* are the capacitance, effective dielectric constant, permittivity of free space, device contact area, and the thickness of dielectric material, respectively.

**Figure 3:**
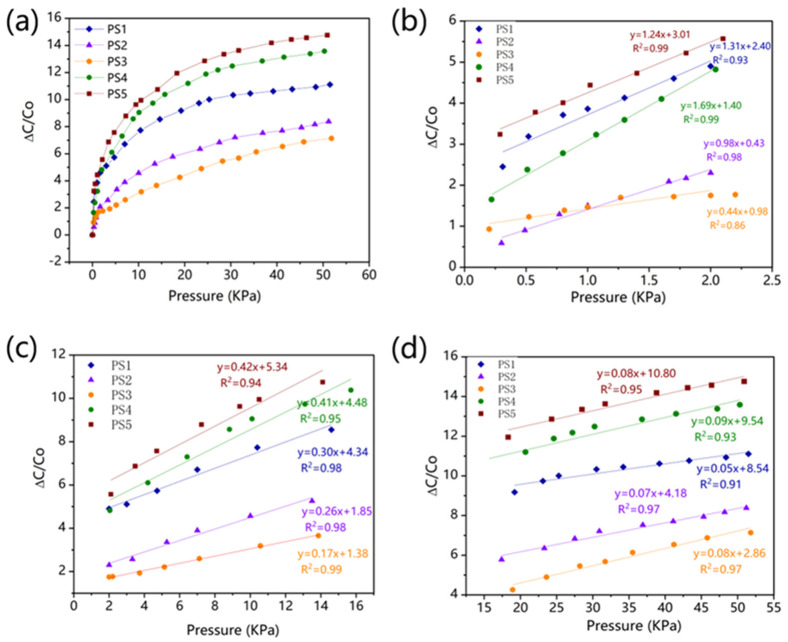
(**a**) Relative capacitance change of pressure sensors PS1–PS5. (**b**) Linear fitting of relative capacitance changes in the pressure range of 0–2 KPa. (**c**) Linear fitting of relative capacitance changes in the pressure range of 2–15 KPa. (**d**) Linear fitting of relative capacitance changes in the pressure range of 15–50 KPa.

When a normal force is exerted on an electrode, the dielectric layer undergoes compression, leading to an increase in capacitance. Consequently, for a fixed overlapping area, the extent of deformation caused by a specific force on the dielectric layer directly influences the distance between the two electrodes, thereby resulting in a significant alteration in capacitance. The determination of sensitivity is elucidated in Equation (2) [21].

(2) $$S=\frac{\partial (\Delta C/C_{0})}{\partial P}$$

where *C* and *C*_0_ are the resultant capacitance and the initial capacitance without loading the pressure (*P*), respectively. Figure 3a displays the curve representing the alteration in relative capacitance of sensors PS1–PS5 as pressure increases. When comparing the three curves of PS1–PS3, it becomes evident that an increase in the height of the micro cone structure corresponds to an increase in sensor sensitivity. The spacing between the electrode plates plays a crucial role in enabling a high aspect ratio for the dielectric layer’s micro-cone, thereby ensuring a heightened level of sensor sensitivity. In comparison to sensors lacking gradient structure dielectric layers (PS1, PS2, PS3), sensors PS4 and PS5 exhibit a more significant relative change in capacitance, suggesting an enhanced sensitivity to some extent through the utilization of gradient structure. The capacitive responses of sensors PS1–PS5 were examined across three distinct pressure ranges: 0–2 KPa, 2–15 KPa, and 15–50 KPa, based on the observed trend in the curve. Under low pressure conditions, the sensitivities of PS4 and PS5 were 1.69 KPa^−1^ and 1.24 KPa^−1^, respectively, with linear correlation coefficients of 0.99 for both. However, the linearity of PS1-PS3, which lack a gradient structure, was less than 0.99. In comparison, PS1 exhibited a higher sensitivity of 1.31 KPa^−1^ due to its excellent aspect ratio of the dielectric layer MCA (A20). As the pressure increased to the range of 2–15 KPa, the sensitivities of PS4 and PS5 became 0.42 KPa^−1^ and 0.41 KPa^−1^, respectively, both surpassing those of PS1, PS2, and PS3 without a gradient structure. Even under high pressure conditions (15–50 KPa), PS4 and PS5 still maintained a certain level of sensitivity. In comparison to the sensors with sensitivity of 2.21 × 10^−6^ KPa^−1^ in previous studies [51], our pressure sensors (PS4 and PS5) with the dielectric layers of gradient structures exhibited considerable higher sensitivity up to 1.69 KPa^−1^.

The dielectric response of the dielectric layer is predominantly influenced by the alterations in contact area under different pressures [25]. As the dielectric layer of the micro cone structure was compressed, the rate of change in contact area gradually diminished, resulting in a decrease in sensitivity. The PS1–PS3, lacking a gradient structure, exhibited remarkable sensitivity at low pressure; however, its sensitivity significantly declined as pressure increased, thereby hindering the attainment of a satisfactory dielectric response across all pressure ranges. Hence, the selection of a gradient structure facilitated the sequential contact of the electrode with the micro cone structure, ensuring a sustained high dielectric response across diverse pressure ranges. The dielectric behavior of GMCA dielectric in distinct pressure regions was influenced by the various types of micro-cone pixels, contingent upon their height gradient.

**Figure 4:**
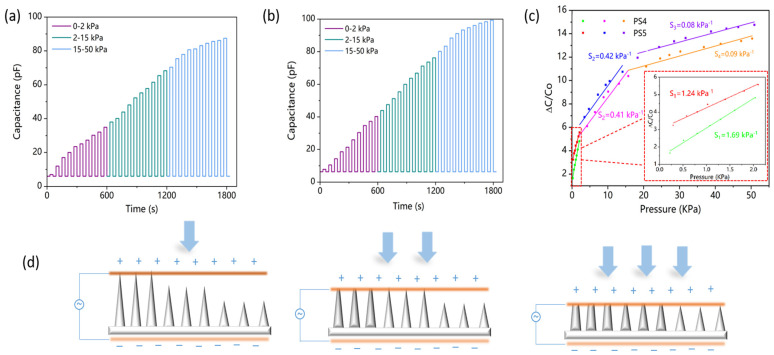
(**a**) Dynamic capacitive response of PS4. (**b**) Dynamic capacitive response of PS5. (**c**) Capacitive sensitivity of PS4 and PS5. (**d**) Working mechanism of the GMCA dielectric.

Under low pressures, micro-cone pixels with comparatively lower height (e.g., B and C) became connected to the air components due to their separation from the upper electrode. As a result, the limited capabilities of pixels B and C significantly constrained the dielectric properties of the GMCA layer, as they were determined by the tallest pixel (pixel A). Following the compression of pixels A, pixels B and C can subsequently come into contact with the upper electrode, thereby transforming their series connection with the air components into a parallel connection. Consequently, in the medium and high-pressure regions (Figure 4d), pixels B and C will exert a dominant influence on the dielectric behavior of the GMCA dielectric.

In principle, changes in the height and number of gradient micro-cone pixels will affect the dielectric behavior of GMCA dielectrics. When no external pressure was applied, a base capacitance of 6pF was measured. The dynamic response of the fabricated pressure sensor (PS4, PS5) for the applied pressure ranges are shown in Figure 4a,b. For PS4, the capacitance of the pressure sensor was increased from the base capacitance of 6 pF to 34.9 pF when the pressure was increased from 0 Pa to 2 KPa, respectively. In addition, it was observed that the capacitance was increased from 34.9 pF to 68.3 pF and 68.3 pF to 87.5 pF when the applied pressure was increased from 2 KPa to 15 KPa, and 15KPa to 50 KPa, respectively. The pressure ranges of 0 KPa to 2 KPa, 2 KPa to 15 KPa, and 15 KPa to 50 KPa resulted in overall relative capacitance changes of 482%, 1038%, and 1358%, respectively. However, for PS4, these values were 538%, 1110%, and 1476%, respectively. Figure 4c demonstrates that PS5, with more high micro cone pixels, exhibited a more significant relative change in capacitance and better overall linearity compared to PS4. Additionally, PS4 demonstrated higher sensitivity under low pressure compared to PS5. Hence, the utilization of PS4 under low pressure conditions can enhance sensitivity, whereas employing PS5 across a broader pressure range can amplify the obtained outcomes. Consequently, PS4 was chosen for subsequent experimentation and implementation in this study.

### 3.2. The Impact of BaTiO_3_ on Sensor Performance and Content Determination

Based on the findings of Equations (1) and (2), enhancing the effective dielectric constant of the dielectric layer can significantly enhance the sensor’s sensitivity. The investigation demonstrated that the inclusion of BaTiO_3_ particles in the composite yielded a substantial dielectric constant. Consequently, the BaTiO_3_-PDMS material was chosen for the fabrication of the capacitors’ dielectric layer in this experiment. Prior research outcomes indicated that the dielectric constant of BaTiO_3_ is contingent upon the crystal’s grain size. The dielectric constant of BaTiO3 was determined to be 1750 for particle diameters ranging from 20 µm to 50 µm, and 5000 for a diameter of 1.1 µm. As the grain size of the BaTiO_3_ crystal decreased, its permittivity value also decreased significantly. When the particle diameter was less than 100 nm, the permittivity value became extremely low [44,57]. Consequently, for this experiment, BaTiO_3_ powder with particle sizes in the micrometer range was chosen as the composite material.

**Figure 5:**
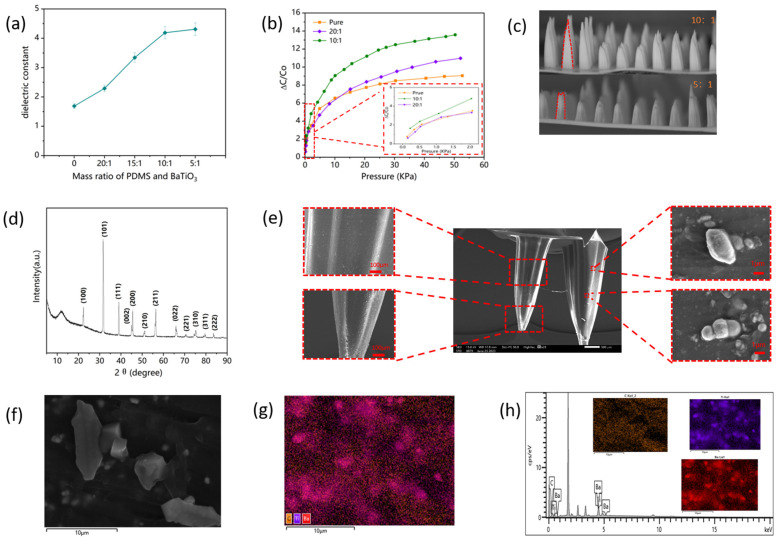
(**a**) Dielectric constant of different PDMS and BaTiO_3_ mixed mass ratios. (**b**) Relative capacitance variation of different PDMS and BaTiO_3_ mixed mass ratios. (**c**) Microscopic images of GMCA dielectric layers prepared at a mass ratio of 10:1 and 5:1 for PDMS to BaTiO_3_. (**d**) The XRD plot of PDMS to BaTiO_3_ at a mass ratio of 10:1. (**e**) The electron microscope scanning image of GMCA with a mass ratio of PDMS to BaTiO_3_ of 10:1. (**f**) The plot of particle size structure of BaTiO_3_. (**h**) The elemental analysis results of Ba, Ti, C. (**g**) The map sum spectrum.

To ascertain the most favorable blending ratio of PDMS and BaTiO_3_ powder, we fabricated BaTiO_3_-PDMS thin films with varying mass ratios and, subsequently, assessed their dielectric constants, as depicted in Figure 5a. As the mass proportion of BaTiO_3_ in the mixture augments, the dielectric constant of the material also increased, thereby suggesting that the proportion of BaTiO_3_ can be maximized to enhance the dielectric characteristics of the dielectric layer. However, the addition of BaTiO_3_ powder in increasing quantities results in a decrease in the fluidity of the mixed solution during preparation, leading to a hardened material that poses challenges in shaping the micro-structure of the dielectric layer. This is illustrated in Figure 5c, where the higher pixel parts in the micro-structure were not formed when a 5:1 mixing ratio was employed. Considering both practicality and the level of difficulty in preparation, we opted for a 10:1 mass ratio of PDMS and BaTiO_3_ to fabricate the dielectric layer GMCA. Figure 5b illustrates the relative capacitance change curves of GMCA pressure sensors fabricated from pure PDMS devoid of BaTiO_3_ addition, as well as composite materials with PDMS to BaTiO_3_ mass ratios of 20:1 and 10:1, correspondingly. The sensitivity of these sensors within the pressure range is documented in Table 2. Evidently, the utilization of a composite material comprising BaTiO_3_ and PDMS exhibited a notable enhancement in sensor sensitivity when compared to pure PDMS materials. This method, which is both simple and easy to operate, deserves attention as a means to enhance the sensor’s sensitivity. Figure 5d illustrates the XRD plot of PDMS to BaTiO_3_ at a mass ratio of 10:1. Additionally, Figure 5e presents the scanning electron microscope image of GMCA with a mass ratio of PDMS to BaTiO_3_ of 10:1. The particle size structure of BaTiO_3_ is depicted in Figure 5f, while the electron microscopy-based elemental analysis results are displayed in Figure 5g,h, showcasing the distribution of Ba, Ti, and C elements.

### 3.3. Hysteresis Response

**Figure 6:**
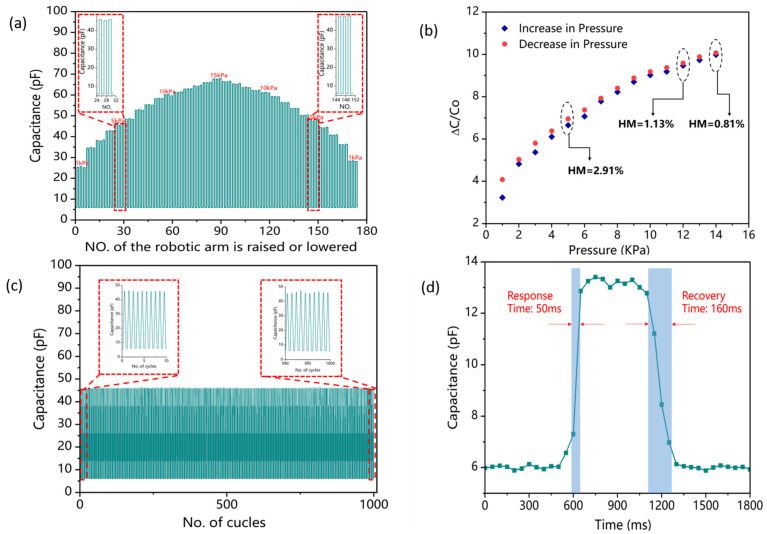
(**a**) Stepwise pressure response of GMCA pressure sensor. (**b**) Hysteresis of GMCA pressure sensor. (**c**) Repeatability of GMCA pressure sensor for 1000 cycles at 5 KPa. (**d**) Response and recovery time of GMCA pressure sensor at 100 Pa of applied pressure.

**Figure 7:**
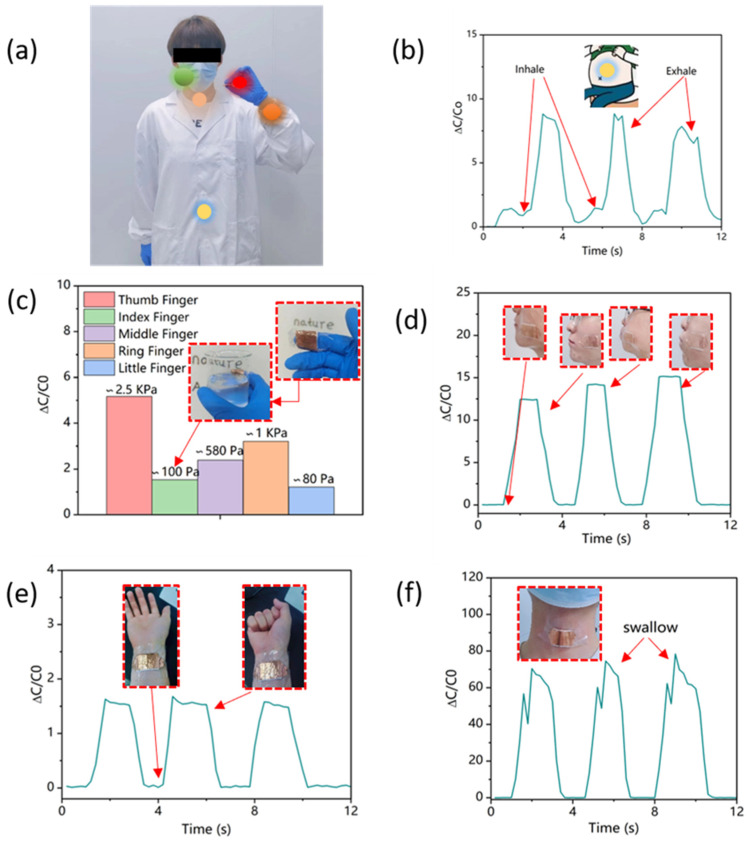
(**a**) Monitoring human motion with the GMCA pressure sensor at different sites. (**b**) The capacitance changes in abdominal pressure during breathing. (**c**) The relative capacitance changes of sensor for thumb, index, middle, ring, and little finger while holding the beaker. (**d**) GMCA sensor recognizes facial expressions. (**e**) Capacitive response of pressure sensor for monitoring hand closing and opening gesture. (**f**) GMCA pressure sensor detects the swallowing movements.

The hysteresis response of the PS4 was investigated for the pressure range of 0 to 15 Kpa. Step-wise pressures increasing from 0 to 15 Kpa and then decreasing from 15 Kpa to 0 Pa, in steps of 1 Kpa, were applied with 3 cycles per step (Figure 6a). The capacitive response of the pressure sensor was measured and the maximum hysteresis (MH) was mathematically calculated using Equation (3) [51].

(3) $$MH(\%)=\frac{[x_{2}-x_{1}]\times 100}{x_{p}-x_{b}}$$

where *x*_1_ and *x*_2_ are the capacitances measured for an applied pressure during stepwise increase and decrease, respectively. *x_p_* is the peak capacitance at 15 KPa and *x_b_* is the base capacitance (at 0 KPa). A *MH* of 2.91% was calculated at an applied pressure of 5 KPa where *x*_1_ and *x*_2_ were measured to be 45.9 pF and 47.7 pF, respectively (Figure 7b). As shown in Figure 6b, a minimum hysteresis of 0.18% was calculated at an applied pressure of 14 KPa (where *x*_1_ = 65.9 pF and *x*_2_ = 66.4 pF). This indicated that pressure sensor has relatively better recovery and elasticity characteristics.

### 3.4. Repeatability

The repeatability test was performed on the pressure sensor for a 1000 loading and unloading cycles of 5 KPa pressure. Figure 6c shows the repeatability of the sensor response; the inset shows the capacitive response of the sensor for 10 cycles between 0–10 and 990–1000 at applied pressure of 5 KPa. It was observed that the capacitance response of the pressure sensor over the 1000 cycles increased from a base value of ~6.0 pF to ~45.9 pF, demonstrating a maximum change of 665 ± 20%. From the results, it can be concluded that the pressure sensor demonstrated high repeatability and durability. It can be inferred that our sensor possessed a robust operational lifespan and was capable of withstanding the conditions associated with multiple repetitive tests in a general usage environment.

### 3.5. Response and Recovery Time

The response and the recovery time of the GMCA pressure sensor was measured by subjecting it to 100 Pa applied pressure. The capacitance of the pressure sensor was increased from 6 pF (base value) to 13.25 pF resulting in a relative capacitance change of 121%. The response time of the pressure sensor (time taken for the capacitance to reach from 10% (T10% = 595 ms) to 90% (T90% = 645 ms) of the total capacitance change (7.25 pF) was calculated to be 50 ms. Similarly, the 100 Pa applied pressure was removed on the pressure sensor and a recovery time of 160 ms was obtained (Figure 6d).

### 3.6. Comparison of the Performance Indicators of Various Microstructure Capacitive Pressure Sensors

Table 3 presents a comprehensive overview of the performance indicators exhibited by diverse microstructure capacitive pressure sensors thus far. The dimensions, aspect ratio, and magnitude of the microstructure play a pivotal role in influencing several performance indicators, such as sensitivity, hysteresis effect, and repeatability. This research endeavor successfully attained heightened sensitivity and reduced response time across a broad pressure spectrum by incorporating high dielectric constant BaTiO_3_ particles and implementing gradient heights. Furthermore, the gradient micro-structure-based sensor developed in this study demonstrated applicability in pressure sensing up to 50 KPa.

### 3.7. Application Demonstration

The potential application of the GMCA pressure sensor in wearable robotics was examined as an application demonstrator. The results, depicted in Figure 7a–f, indicate that GMCA pressure sensors have the ability to detect various human movement behaviors, including facial expressions, breathing, grasping heavy objects, swallowing, and more. Specifically, Figure 7b displays the alterations in abdominal pressure during breathing. To measure these changes, the GMCA sensor was affixed to the abdomen of a volunteer who then performed breathing exercises. The compression of the sensor during exhalation and inhalation allowed for the measurement of corresponding changes in capacitance. Hold a beaker containing water using a finger connected to a GMCA pressure sensor and measure the alteration in its capacitance. Figure 7c illustrates the variation in capacitance response among different fingers, revealing that the thumb, when gripping a heavy object, exhibited the greatest change, suggesting that it experienced the highest pressure. The capacitance of the GMCA pressure sensor fluctuated rapidly and then stabilized as facial expressions altered (cheeks protrude), remaining relatively constant. Once the expression returned to its original state, the capacitance promptly reverted to its initial value (Figure 7d). The GMCA pressure sensor was affixed to the wrist of participants in order to monitor the capacitance response when performing hand opening and closing gestures. As depicted in Figure 7e, the observed change in capacitance during these gestures corresponded to a pressure of approximately 0.1 KPa. Furthermore, the GMCA pressure sensor was capable of detecting swallowing movements, with the capacitance changes observed during three distinct swallowing movements being largely consistent (Figure 7f). The experimental findings presented herein showcase the extensive potential of GMCA pressure sensors in the domains of biosensing and wearable sensing devices for human–machine interfaces.

## 4. Conclusions

This study presented a novel capacitive pressure sensor based on laser-assisted engraving technology, referred to as GMCA sensor. The dielectric layer of the sensor was fabricated using BaTiO_3_-PDMS material with a gradient structure, aiming to enhance the sensitivity and linearity of the sensor. Furthermore, the impact of incorporating BaTiO3-mixed PDMS material on the performance of the sensor was investigated, and a mass ratio of 10:1 was identified as the optimal configuration. The performance of the GMCA pressure sensor was evaluated by subjecting it to a range of pressures from 0 to 10 KPa. The pressure sensor demonstrated a sensitivity of 1.69 KPa^−1^, 0.41 KPa^−1^, and 0.09 KPa^−1^ within the pressure ranges of 0 Pa to 2 KPa, 2 KPa to 15 KPa, and 15 KPa to 50 KPa, respectively. The utilization of flexible PDMS facilitated the pressure sensor to showcase notable attributes such as a rapid response time of 50 ms, minimal hysteresis, a recovery time of 160ms, as well as exceptional repeatability and durability across a broad pressure spectrum. The findings indicate that the GMCA pressure sensor, which utilized a gradient structure and BaTiO_3_-PDMS material, exhibited notable sensitivity and a broad linear pressure range. These outcomes underscore the adaptability and viability of this technology, thereby enabling enhanced flexibility in pressure sensors and fostering advancements in laser manufacturing and flexible devices for a wider range of potential applications. Furthermore, it is anticipated that our GMCA-based capacitive sensor design holds potential for utilization as a wearable sensor for health monitoring and noninvasive detection on the skin, offering promising strategies for future applications. Subsequent investigations will encompass the examination of environmental variables, such as humidity and temperature, and their influence on the performance of the sensor.

## Figures and Tables

**Table 1:** Dielectric layer and its main mode engraving parameters.

Sensor Number	Dielectric Layer	Laser Parameters		Main Module	Micro-Structures Height (μm)
		Speed (mm/s)	Power (%)		
PS1	MCA(A_20_)	100	30	M_1_	4500
PS2	MCA(B_20_)	100	40	M_2_	3500
PS3	MCA(C_20_)	100	50	M_3_	2500
PS4	GMCA(A_3_B_6_C_11_)	100	30, 40, 50	M_4_	4500
PS5	GMCA(A_2_B_5_C_13_)	100	30, 40, 50	M_5_	4500

**Table 2:** Sensitivity of sensors with different mixing ratios of dielectric layers.

Mass Ratio of PDMS and BaTiO_3_	0–2 Kpa	2–15 Kpa	15–50 Kpa
pure	1.30	0.17	0.04
20:1	1.37	0.27	0.08
10:1	1.69	0.41	0.09

**Table 3:** Summary of the performance indicators of various microstructure capacitive pressure sensors.

Micro-Structure	Fabrication Method	Mechanism	Pressure Range	Sensitivity	Response Time	References
Tilted micropillar	Photolithography	Capacitive	0–40 KPa	0.42 KPa^−1^	70 ms (8 KPa)	[58]
Semi sphere	Electroless plating	Capacitive	0–10 KPa	0.13 KPa^−1^	–	[59]
Micro-square	Photolithography	Capacitive	0.5 Pa–3 KPa	0.185 KPa^−1^	–	[60]
Micro-porous	Chemical method	Capacitive	<0.02 KPa	1.18 KPa^−1^	150 ms (0.6 KPa)	[61]
Micro-pyramid	Laser patterning	Capacitive	0–0.1 KPa 0.1–1 KPa 1–10 KPa	0.221% Pa^−1^ 0.033% Pa^−1^ 0.011% Pa^−1^	50 ms (0.02 KPa)	[51]
Gradient micro-cone	Laser engraving and molding	Capacitive	0–2 KPa 2–15 KPa 15–50 KPa	1.69 KPa^−1^ 0.41 KPa^−1^ 0.09 KPa^−1^	50 ms (0.1 KPa)	This work

## Data Availability

Data will be made available on request.
